# Making sense of shaky data in humanitarian crises

**DOI:** 10.3389/fpubh.2025.1602366

**Published:** 2025-06-16

**Authors:** Sandro Colombo, Chiara Altare

**Affiliations:** ^1^Independent Consultant, El Escorial, Spain; ^2^Johns Hopkins Bloomberg School of Public Health, Baltimore, MD, United States; ^3^Johns Hopkins Center for Humanitarian Health, Baltimore, MD, United States

**Keywords:** humanitarian crises, data interpretation, decision-making, epidemiology, uncertainty

## Abstract

Humanitarian decision-making occurs in volatile and politically charged environments where information is often incomplete, outdated, or conflicting. Effective humanitarian response often requires interpreting poor-quality data to guide interventions, allocate resources, and assess impact. Despite advances in evidence generation, knowledge gaps persist, and decisions are frequently influenced by political and organizational factors rather than by data. This paper argues that data interpretation is an area of weakness in humanitarian response. Data availability and quality vary across crises, with methodological challenges and political sensitivities further complicating interpretation. The three examples of Darfur (Sudan), Yemen and Ethiopia illustrate how conflicting information and ambiguous interpretation can negatively impact critical decisions with far-reaching consequences on the affected communities. This paper concludes with suggestions for making better interpretation and use of data in humanitarian crises.

## 1 Introduction: every major decision in humanitarian crises carries a degree of uncertainty

*“All meanings, we know, depend on the key of interpretation.”* (1)

Humanitarian actors operate in highly politicised environments often marked by chaos and volatility. They must manage incomplete, inaccurate, and quickly outdated information and coordinate with numerous agencies, each pursuing their agendas. Politicians, managers, and humanitarian workers make a variety of decisions, such as whether to initiate, scale up, or scale down a response; which interventions to prioritise; how to allocate financial, technical, and human resources; whether and how to target the most vulnerable population groups, and how to adjust the response based on monitoring the crisis’s evolution and the effectiveness of operations (2). Some of these decisions—or the lack thereof—can have significant negative and long-lasting consequences on affected populations. For example, misinterpreting or overlooking available information—even when incomplete or of poor quality—can lead to underestimating the severity of a crisis, neglecting priority needs, and ignoring human rights abuses. This, in turn, may result in reduced humanitarian aid, weakened advocacy, ineffective response strategies, and inadequate interventions—ultimately exacerbating the suffering of affected populations and increasing morbidity and mortality. Furthermore, interpreting crisis situations through universal standards and thresholds, with the implication that indicators and benchmarks are equally applicable across diverse contexts and cultures, has resulted in tensions among humanitarian organizations, exemplified by the criticisms levelled against the Sphere Project (3).

Evidence—defined as “*any form of knowledge, including but not limited to research, that is of sufficient quality to inform decisions*” (4)— is essential for guiding action. Despite the increase in research studies in humanitarian settings over the last decades and the generation of critical evidence in key sectors (5, 6), knowledge gaps remain. The existence of evidence does not ensure, however, that evidence-based decisions are made. Political considerations and organisational aspects influence decisions, sometimes regardless of, or even contrary to, the available information. In addition, past knowledge and experience, assumptions, established narratives, and prior decisions (e.g., on budget allocation or prioritisation of some population groups) restrict the available response options. Moreover, the quality of evidence can be compromised by security constraints, data collection challenges, biases, and partisan manipulation. Insecurity or political barriers may prevent humanitarian workers from gathering critical information. Some evidence can also be withheld when its disclosure might harm political agendas. As a result, conflicting information and gaps in understanding are common, making it difficult to make sense of data and inform effective decisions.

Interpreting crises is not solely reliant on hard data. Insights from key informants, shaped by their experience in similar contexts and underlying assumptions, also play a crucial role in understanding the crisis and guiding the response. Experience is particularly valuable when decisions must be made urgently, the information available is inadequate, and the situation is familiar to the decision maker (7). Indeed, the cost of delaying decisions to obtain more and better data can be high in terms of increased suffering and lives lost. Yet, previous experience is part of the evidence to use, as most crises’ effects can be anticipated, especially when knowing the context where the crisis strikes. However, just as poor-quality data can lead to misinterpretation, overconfidence in intuitive choices and experience, cognitive biases, and neglect of background information can result in misunderstanding the situation. In complex contexts, interpretation inevitably involves some degree of subjectivity in data selection, assessment of their quality, and analysis assumptions: “*There is no objective measure for the subjective components of interpretation*” (8). Consequently, the risk of interpretive biases is a constant concern.

This article argues that data interpretation is a weakness in humanitarian response. It emphasizes the importance of not accepting the data at face value but scrutinising them and understanding their limitations, recognising the importance of contextual factors, and integrating local knowledge to interpret data effectively. We first highlight key issues related to data availability and quality in humanitarian settings and we then provide three examples of interpretation challenges in selected domains of public health information: mortality, malnutrition and famine, and health service delivery. We conclude by sharing suggestions on interpreting data more effectively, when understanding is complex due to conflicting facts, information gaps, or discrepancies between data sources.

## 2 Data availability and quality

“We are absolutely sure that we cannot be sure about the data” [Rosling, H., 2015, cited in (9)]. Data availability and quality vary extensively across crises and countries, although numerous initiatives have strengthened data collection methods and systems in humanitarian settings over the last decades. Such efforts include, but are not limited to, the SMART methodology for nutrition and mortality assessments (10), the District Health Information System (DHIS2) for routine health data management at district level (11), innovative approaches to estimate mortality that aim to overcome the complexity of collecting data in challenging settings (12) maternal death and perinatal surveillance and response (13), as well as emergency disease surveillance systems (14). Besides providing guidelines and standard operating procedures, these initiatives have trained thousands of health practitioners across crises, and several of these systems are now part of the backbone of the health information systems in many countries. Yet, the lack of financial and human resources limits such complex data systems’ functionality, coverage, and completeness.

Quality standards and related assessment methods exist, yet they are not routinely used. Assessing data quality, essential for accurate interpretation, involves expert knowledge of data collection and analysis methods and their limitations, and understanding the challenges faced in the specific humanitarian context. Irrelevant or flawed data—lacking sources, derived from poor methods, or missing a discussion of methodological limitations—should be excluded from analysis only after thorough scrutiny. The risk of over-interpreting poor-quality data must be balanced against ignoring them too quickly, which could lead to overlooking unexpected findings and important clues. Understanding the context in which data were collected is essential for determining whether their quality is sufficient for use. For example, insecurity may hinder data collection in inaccessible areas or restrict it to ‘quick and dirty’ methods. In such cases, the only available options may be qualitative information from relevant informants (maybe even conducted remotely) combined with realistic assumptions about the situation and the unknown humanitarian needs. Data deemed plausible and reliable should be retained for further analysis and triangulation with other sources and methods. Filtering out reliable data from existing information is often challenging, given the wide range of indicators from diverse sources and methods and in assorted formats typically available during a crisis.

## 3 Making sense of scarce data

### 3.1 Mortality

Armed conflicts result in injuries, disabilities, and excess morbidity and mortality, especially when humanitarian assistance is inadequate, and health systems have collapsed. A rise in mortality is a crucial indicator of the severity of a crisis. Monitoring mortality, along with other selected indicators, can contribute to assessing the overall effectiveness of the response. It also serves to document human rights violations, hold warring parties accountable, advocate for protecting civilians in armed conflicts, and enhance the crisis’ visibility among international actors and donors. Yet, the death toll is one of the most sensitive and politically charged metrics to estimate during conflict (see Box 1). From a methodological perspective, estimating mortality is fraught with challenges (15), including multiple potential sources of biases and lack of capacity in the design and supervision of data collection and analysis.

BOX 1 **Discordant mortality data in Darfur.**

Sudan has been one of the most conflict-ridden countries for several decades. In 2003–2004, mass atrocities carried out in the Darfur region by the Janjaweed militia supported by the Al-Bashir government shocked the world. In this politicised context, mortality estimates became extremely sensitive. Dozens of mortality surveys, most of them at a small geographic scale, were conducted in the region. One of these, supported by the World Health Organization in 2004, concluded that the crude mortality rate in the northern and western Darfur states was above the emergency threshold (16). A nutritional and mortality survey conducted by the World Food Programme in the same period and region reached the opposite conclusions, estimating that mortality was below emergency benchmarks (17). The WHO survey only covered IDPs, whereas the WFP survey included both IDPs and crisis-affected residents. Insecurity negatively affected the conduct of the two studies: WHO could only cover an IDP camp in South Darfur, whereas WFP had to limit the mortality assessment only to secure areas in the North Darfur. In the WFP survey, the mortality estimate was higher among IDPs than among residents, but the difference was not statistically significant. Both surveys used the same two-stage cluster sampling methodology, with a similar number of clusters for each mortality estimate. WHO, however, conducted three surveys to obtain separate mortality estimates for each of the three states, whereas WFP averaged the estimate for the whole Darfur. Finally, the recall period was different: June–August 2004 for WHO and February–September 2204 for WFP. The discrepancy of results is likely to be attributable to the different population studied and the diverse recall periods. The higher mortality among displaced populations than among residents and, possibly, a higher insecurity and impact of the conflict in the shorter period covered by WHO, might account for the difference. This example highlights that data interpretation may be challenging, especially for readers unfamiliar with the methodological aspects and inherent limitations of mortality studies in crisis settings. The conflicting picture presented by these and other inconsistent findings led the Washington Post to refer to mortality data in Darfur as a “statistical anarchy” (18).

### 3.2 Malnutrition and famine

Climate change and conflicts are the key drivers of current food crises. In recent years, food crises have reached catastrophic levels with extremely high mortality in countries affected by conflicts, such as South Sudan, Sudan, Yemen, Ethiopia, Somalia, and the Gaza Strip. In these contexts, intentional starvation of civilians has been used as a tool of war (19).

A classification system of food insecurity—The Integrated Phase Classification (IPC) (20)—provides a rigorous, evidence- and consensus-based analysis framework to assess the severity of the crisis for early warning and real-time analysis. The IPC is based on multiple food insecurity, malnutrition, and mortality indicators. For example, a famine (i.e., phase five, the worst category of the IPC scale) is characterised by a crude death rate greater than 2 per 10,000 people per day, a prevalence of global acute malnutrition greater than 30%, and more than 20% of households facing extreme food deficits. For a situation to be classified as a famine, all three indicators must reach the specified thresholds; otherwise, it is categorised as an emergency or catastrophe. The IPC relies on a data-driven approach, requiring rigorous data collection and analysis. As challenges remain in applying this approach in data-scarce settings (see Box 2 on Yemen), officially declaring a famine remains complex and politically charged, although special additional protocols have been adapted for areas with limited or no humanitarian access (21). Indeed, governments may oppose an official declaration of famine that would carry the stigma of their inability to feed their people or purposely starve them in a civil war, as in the Tigray region of Ethiopia in 2021 (22).

Interpreting food insecurity based solely on severity is, however, insufficient. The magnitude and duration of the crisis also contribute to the public health and social consequences of hunger. Excess mortality can be significantly high even in crises that do not reach the severity of the famine phase of the IPC, especially in protracted food insecurity crises (26) (Box 2).

BOX 2 **Was there a famine in Yemen in 2019?**

The Yemen crisis, once regarded as one of the world’s worst emergencies and the largest relief operation, had faded away from public attention, overshadowed by other crises.

During the 2018–19 crisis peak, with a blockade of food and humanitarian aid, Save the Children warned that the country was on the brink of the “worst famine in 100 years,” a statement confirmed by the top UN humanitarian official (23). In contrast, Médecins Sans Frontières (MSF) disputed this characterization, stating, “There is no quality data available to declare that a famine is imminent. Calling the humanitarian crisis in Yemen ‘the worst in the world’ is not only most likely incorrect but also inept, given how inflated the diagnoses seem” (24, 25).

However, failure to meet all three IPC famine thresholds does not imply that thousands are not dying from starvation or hunger-related diseases, particularly during prolonged food crises. In South Sudan, the majority of excess deaths between 2014 and 2018 occurred in phase three and four.

### 3.3 Immunisation coverage: conflicting findings

Children in crisis-affected countries are at increased risk of excess morbidity and mortality from vaccine-preventable diseases due to the disruption of preventive and curative health services, food insecurity, and unsanitary living conditions, to name a few risk factors.

Immunisation is a key public health intervention to mitigate excess morbidity and mortality in crises. Therefore, assessing and monitoring its coverage is crucial for designing an effective humanitarian response strategy and targeting the most vulnerable children. Administrative data from the routine health information system, small-scale cluster surveys, and country-wide demographic surveys are the main approaches for estimating and monitoring immunization coverage over time (Table 1).

**Table 1:** Approaches for estimating and monitoring immunization coverage: key advantages and disadvantages.

Approach	Advantages	Disadvantages
Administrative data (often using DHIS2)	Real-time local monitoring and action if coverage declines Possible data disaggregation at the health facility level Part of the routine reporting system at the facility level	Potential transcription/input errors; possible incomplete reporting due to limited resources Denominators are seldom available or imprecise and may not include population movements Set targets can be unrealistic Data can be inflated when linked to performance-based incentives Wastage of doses is sometimes ignored, leading to overestimation
Small scale surveys	WHO guidance and statistical software available They are relatively inexpensive and quick Immunization data can be collected along with nutrition and/or mortality data Sampling design can be simplified especially in small IDP or refugee camps or adapted (Lot Quality Assurance Sampling^a^) (28)	Information bias when vaccination cards are unavailable, and data are collected through verbal history It requires supervision, logistic support, and financial resources Cluster sampling design prevents data disaggregation and impacts the sample size Accessibility to populations can be limited in insecure areas They may not be representative of the broader situation
Demographic surveys (Demographic Health Survey, Multiple-Indicators Cluster Survey)	Several indicators can be collected in addition to immunisation Standard methods and questionnaires available	Costly and of long duration Usually conducted every 5 years, not suitable for humanitarian crises Emergency settings are not always included

a Lot quality assurance sampling is a simplified sampling approach used to classify performance of humanitarian response at the local level as “adequate” or “inadequate” according to a set of pre-established decision rules.

Comparing immunisation coverage estimates from different approaches should be conducted cautiously, as the indicators can refer to different population groups and reflect the limitations of each method (see Box 3). In humanitarian crises, weakened health information systems, limited access to health services, and population movements can exacerbate existing challenges to quality immunization data, such as imprecise denominators, target overreliance, and workforce capacity gaps (28). Triangulating immunization coverage data from different methods may reveal inconsistencies. In such cases, informants’ perspectives help explain the discrepancies and identify the most reliable source to use.

BOX 3 **When there is no golden standard for the estimation of immunisation coverage.**

A recent study analysed the immunisation coverage and data quality in three regions of Ethiopia in 2023 following the health system disruption caused by COVID-19, internal conflict, and displacement (29). Two-thirds (66.4%) of the children were fully vaccinated by 12 months of age, as reported through health facility administrative reports. The study also showed poor quality of immunization data, determined by comparing DHIS2 reports against facility registers and tally sheets, the source documents. DHIS2 data were found to be higher than the registers, with a 6–16% discrepancy between the two sources. Informants stated that some providers focused on providing vaccination services at the expense of recording the data. They also suggested that data could have been manipulated to give a false impression of achievement.

## 4 Recommendations

The examples discussed in this paper illustrate that data interpretation can be challenging, particularly in crises marked by complexity and uncertainty where relevant information is missing, or discrepancies exist between indicators from different sources. In such situations, there is no fixed template for interpretation, rather we offer the following recommendations.

For practitioners:

- The initial interpretation should consist of hypotheses about what the data mean, taking into consideration trends, patterns, thresholds, seasonality, groups vulnerabilities. These tentative hypotheses must then be confirmed or rejected as new evidence emerges, expert opinions are collected, and alternative explanations are considered. However, when life-and-death decisions must be made swiftly, experience from relevant past crises and evidence of effective interventions become crucial: “The art of relief is to make hard decisions under pressure and with minimal information” (9, 30).
- Seemingly implausible data that challenge initial intuitions and assumptions should not be immediately dismissed, as they may offer valuable insights and prompt a reassessment of the data: “outliers cannot be ignored” (31). This entails that humanitarians identify and question their own confirmatory biases, and apply the same level of critical scrutiny to data that support their beliefs as they do to findings that challenge their assumptions and expectations. Triangulation of different sources and combination of quantitative and qualitative methods can help fill information gaps, check assumptions, and interpret uncertain data. The example of immunisation coverage in Ethiopia (Box 3) illustrates, however, that the comparison of data from various sources and methods requires an in-depth understanding of the methodologies used to examine the specific threats to validity associated with each data type and source.
- Interpretation is always context-dependent: “Evidence informs aid policy and practice only when the political context, the networks, and the knowledge are all in alignment” (31). Understanding the overall political, social, military, and cultural context is especially important in protracted humanitarian crises, in which a purely relief-based approach may be neither appropriate nor sustainable.
- Qualitative insights from multiple sources, such as affected communities, service providers, and colleagues from other organizations, can shed light on aspects of difficult interpretation. For example, assessing and quantifying needs can be contentious, as agencies may prioritise needs aligning with their own mandates rather than with the perspectives of affected communities (3). Qualitative information is also relevant for capturing intangible elements that are relevant to the humanitarian response, such as the health-seeking behavior of affected communities and the acceptability of specific public health interventions. For example, the 2013–2016 response to the Ebola epidemic in west Africa has shown the importance of understanding the sociocultural and political context and of engaging with the affected communities to build trust.
- Information should be collected, analysed, and interpreted correctly and communicated effectively, especially to decision-makers who may lack familiarity with the crisis and methodological expertise: “The way in which information is presented can be crucial to its uptake and use by decision-makers” (32). Effective communication ensures that data and their interpretation are used to inform, not persuade decision-makers; that uncertainties are communicated transparently; and that the quality of the evidence is clearly stated.

For decision-makers:

- Information is often incomplete and imperfect during the acute phase of a crisis. Therefore, careful attention must be given to how uncertainty and data limitations impact the interpretation and ultimately shape decision-making. Decision-makers may not all be competent in interpreting poor-quality data in a complex context. Consequently, they must rely on the judgment of experts who have the necessary methodological background and experience in humanitarian crises.
- “To understand is to interpret” (33). Straightforward decisions on humanitarian action are possible when robust data are available, evidence is clear, and resources for effective interventions are in place. However, when data are uncertain, contexts complex, and political pressures high—as illustrated by the examples in this article—understanding a crisis demands collaborative interpretation among humanitarian actors, even when disincentives to information sharing are present (34).

## 5 Discussion and conclusions

Data do not always speak for themselves. The examples in this article underscore the need for greater attention to data interpretation. The controversy surrounding the occurrence of famine in Yemen highlights that a lack of evidence does not necessarily imply evidence of absence. Famine is not a sudden, binary event but a gradual process of worsening food insecurity, during which thousands can die from starvation and related causes without a famine being declared.

The conflicting findings of the two mortality surveys in Darfur can be understood only by examining differences in the populations studied, recall periods, and possibly the timing of violence peaks. However, politicians, journalists, and activists are not always equipped to interpret the findings in light of such methodological nuances. Better coordination among the agencies that designed and conducted the surveys could have enhanced the efficiency of the studies and improved the consistency of the results, thereby reducing confusion.

The discrepancy between routine and survey estimates of immunisation coverage in Ethiopia underscores the need for caution in the interpretation of findings from triangulation of different sources. Indeed, both administrative and survey data are susceptible to shortcomings and errors, which, in the Ethiopia case study, were understood through key informants’ interviews. As outlined in our recommendations, qualitative information can add depth to interpretation by shedding light on aspects that may not be immediately apparent.

Humanitarian crises—particularly conflicts—are inherently unstable and chaotic, with outcomes that are often unpredictable. They stand in stark contrast to the controlled environments typically required for rigorous data collection, analysis and interpretation (35). Crises therefore call for honest interpretation of poor-quality data and complex contexts—interpretation that acknowledges its limitations, while drawing on local knowledge and lessons learned from past crises. Oversimplified and flawed narratives, often presented by news media, can distort public understanding, misinform decision-makers, and ultimately contribute to ineffective or even harmful responses.

“When reporters do not know what is happening, remarked the journalist Hilsum, they call it anarchy. And when aid workers do not know what is happening, Duffield added, they call it a complex emergency” (36). Uncertainty is a constant attribute of humanitarian crises, with both “known and unknown unknowns” -in the Rumsfeld’s categorisation-confronting humanitarian actors. Efforts aimed at reducing it should continue by leveraging new technologies, sustained analytical capacity strengthening and advocating for humanitarian access. Given recent effective and announced funding cuts (37), all efforts to manage uncertainty are more necessary than ever to make the best use of available information for improved humanitarian action.

## Data Availability

The original contributions presented in the study are included in the article/supplementary material, further inquiries can be directed to the corresponding author.
